# Viability and collagen secretion by fibroblasts on titanium surfaces with different acid-etching protocols

**DOI:** 10.1186/s40729-019-0192-4

**Published:** 2019-11-21

**Authors:** Vilton Zimmermann de Souza, Rafael Manfro, Júlio César Joly, Carlos Nelson Elias, Daiane Cristina Peruzzo, Marcelo Henrique Napimoga, Elizabeth Ferreira Martinez

**Affiliations:** 1 Division of Implantology, Faculdade São Leopoldo Mandic, Campinas, SP Brazil; 2 Division of Implantology, SOEBRÁS, Passo Fundo, RS Brazil; 3 Materials Science Department, Instituto Militar de Engenharia, Rio de Janeiro, RJ Brazil; 4 Division of Immunology, Faculdade São Leopoldo Mandic, Campinas, SP Brazil; 5 Division of Oral Biology and Cell Biology, Faculdade São Leopoldo Mandic, Rua José Rocha Junqueira, 13, Campinas, SP 13045-755 Brazil

**Keywords:** Surface treatment, Dental implant, Connective tissue

## Abstract

**Background:**

From the consolidation of surface treatments of dental implants and knowledge on the cellular mechanisms of osseointegration, studies have highlighted the importance of a connective tissue seal against the implant to prevent contamination from the oral environment and consequent biofilm formation.

**Objective:**

This *in vitro* study aimed to evaluate whether different titanium surface treatments using acid solutions promoted an increase in collagen secretion, proliferation, and viability of fibroblasts.

**Material and methods:**

Commercially pure grade-4 titanium disks (6 × 2 mm) were treated with different acid solutions (hydrochloric, nitric, and sulfuric) for 20 and 60 min, respectively, obtaining mean surface roughness of 0.1 to 0.15 μm and 0.5 to 0.7 μm. Human fibroblasts were seeded onto different surfaces and assessed after 24 h, 48 h, and 72 h for cell proliferation and viability using Trypan blue staining and MTT, respectively, as well as the secretion of type I collagen on to such surfaces using ELISA. Machined titanium surfaces were used as controls. Data were statistically analyzed using one-way ANOVA and Fisher's LSD test for multiple comparisons, adopting a significance level of 5%.

**Results:**

No significant difference was observed in cell proliferation for the different surfaces analyzed. Cell viability was significantly lower on the machined surface, after 48 h, when compared to the groups treated with acid for 20 or 60 min, which did not differ from each other. The expression of type I collagen was lowest on the acid-treated surfaces.

**Conclusion:**

The results showed that the acid treatment proposed did not promote fibroblast proliferation and viability nor favor type I collagen synthesis.

## Introduction

Consolidation of surface treatments for dental implants and knowledge on the cellular mechanisms of osseointegration has propelled research on the sealing capacity of bone to implant surfaces. Although osseointegration is extremely important for implant success, biological sealing of the perimplantar connective tissue is crucial to maintain success in the long-term because it acts as a first barrier to epithelial migration [1].

Collagen fibers from the band of connective tissue adjacent to the junctional epithelium, which would be directed perpendicularly to the tooth surface, are directed parallel to the long axis of the implant, forming a belt-like structure around the cervical portion of the implant [2–4]. Therefore, no connective tissue fiber attachment is established with the implants, but only direct contact of fibroblasts from this region with the titanium surface, which together with low vascularization favors bacterial penetration [3, 5, 6].

There is no consensus in the literature as to whether surface treatment of prosthetic abutments promotes increased fibroblast proliferation and adhesion. Some studies have shown, however, that topographic modifications to the surface of prosthetic abutments maybe beneficial to the connective tissue adjacent to the abutments, optimizing repair of the gingival tissues [7–11]. Nanotopography and macrogeometry may influence cell adhesion and behavior, which may contribute to such findings.

Considering the importance of a marginal seal for the health and quality of the perimplant tissues, understanding fibroblast behavior on different surface features is fundamental to control the progression of perimplant disease, tissue stabilization, and esthetics. Therefore, the aim of this study was to evaluate the effect of titanium surface treatment using different acid etching time schedules on the morphology, proliferation, and viability, as well as collagen synthesis by gingival fibroblasts.

## Material and methods

This study was approved by the Research Ethics Committee of the São Leopoldo Mandic Research Institute, Campinas/SP (protocol No. 59866216.6.0000.5374). Titanium disks were commercially pure, grade 4 (*n* = 108), measuring 6 mm in diameter by 2 mm in thickness, provided by the company *Conexão Sistemas de Próteses* (Arujá, São Paulo).

**Fig. 1:**
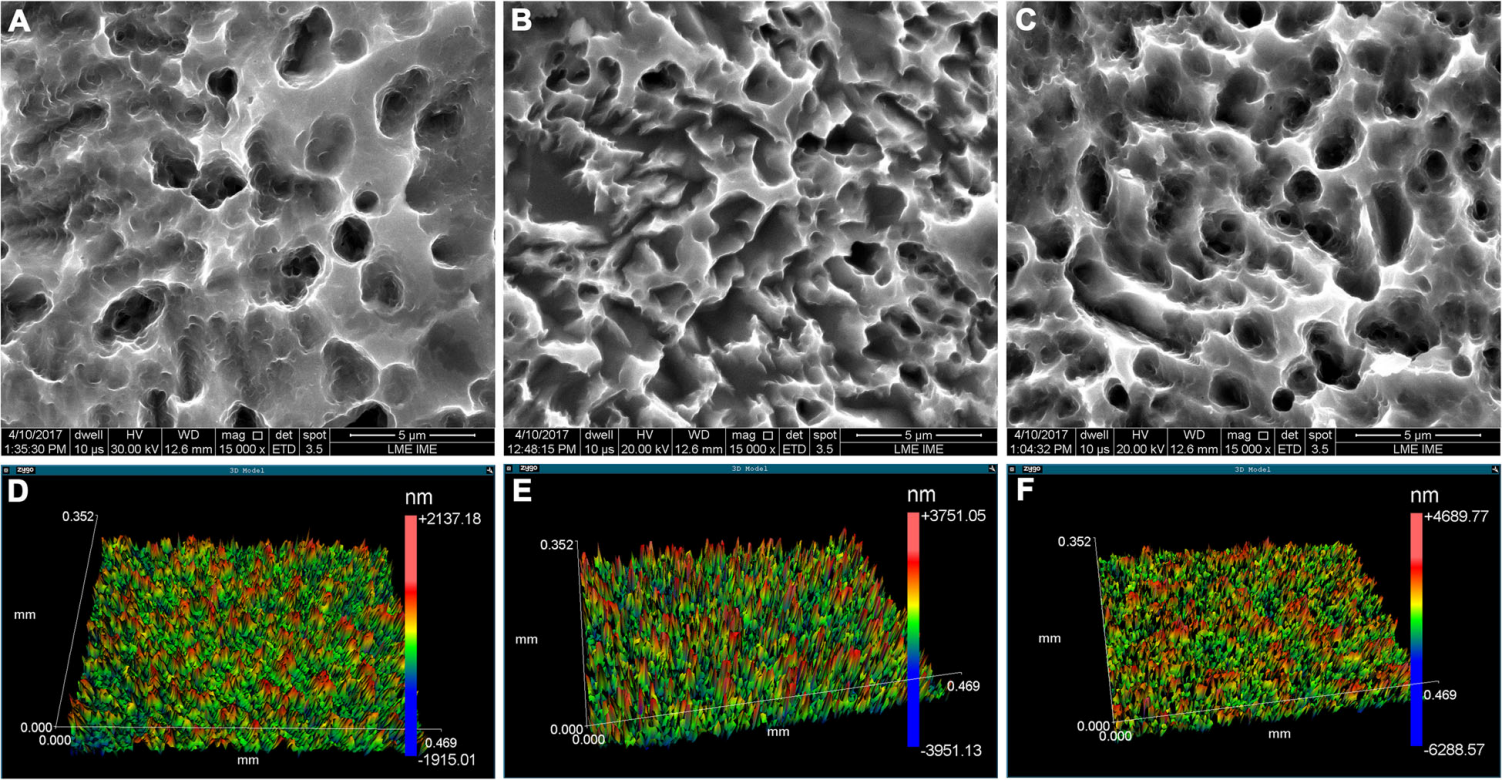
Scanning electron microscopy and laser interferometry. **a**, **d** Machined surface. **b**, **e** 20-min acid treatment. **c**, **f** 60-min acid treatment. Original magnification, × 15,000

For the treatment of titanium disk surfaces, sulfuric, nitric, and hydrochloric acid solutions were used for 20 min (*n* = 36) and 60 min (*n* = 36). As a control, disks with no surface treatment (*n* = 36, machined surface only) were used. The concentration of each acid in the solutions is not described because the company did not wish to disclose it. Roughness was measured using a contact profilometer (Mitutoyo, Surftest model SJ200, Brazil, Suzano). Four linear measurements were performed on each sample according to DIN ISO 1302 standard, and the arithmetic mean of the absolute values for each disk (Ra) was calculated. The average roughness (Ra) obtained was 0.1 to 0.15 μm, for 20 min of acid treatment, and 0.5 to 0.7 μm, for 60 min of acid treatment. Figure 1 shows the morphological characteristics of the surfaces obtained.

The disks were sterilized in ethylene oxide (Acecil, Campinas, São Paulo) and used for the subsequent experiments. Three different human fibroblast cell lines were obtained from the Cell Bank of the Cell Culture Laboratory (São Leopoldo Mandic Research Institute, Campinas) to control for phenotypic and genotypic variations. These cells were previously isolated by primary culture of human gingiva from three patients [12].

### Cell proliferation and viability assays

The cells were cultured on 96-well plates (Corning, NY, USA) at an initial concentration of 110 cells/mm^2^. For the cell proliferation assay, the fibroblasts were detached using 0.05% trypsin and counted on a hemocytometer to calculate proliferation indices at 24 h, 48 h, and 72 h. Cell viability assay was evaluated by 3-[4,5-dimethylthiazol-2-yl]-2,5-diphenyl tetrazolium bromide (MTT, Sigma), at the same time periods. The optical density was read at 570–650 nm on a plate reader (Epoch, Bio-Tek, Winooski, VT), and data were expressed as absorbance.

The experiments were repeated three times, under the same conditions, to ensure accuracy.

### Type I collagen quantification (ELISA)

Quantification of secreted type I collagen by fibroblast cultures on to the different surfaces was evaluated by enzyme-linked immunosorbent assay (ELISA). The supernatant was collected and centrifuged at 336*g* for 10 min. Type I collagen quantification was carried out using the Human Type I Collagen kit (R&D Systems, Minneapolis, USA) according to the manufacturer’s instructions. The values were expressed as picogram per milliliter. All the experiments were carried out in triplicate.

### Statistical analysis

To investigate whether acid treatment time had an influence on cell proliferation and viability and type I collagen expression after 24 h, 48 h, and 72 h of culture, one-way ANOVA analysis was applied. Fisher’s LSD test was used for multiple comparisons. Statistical calculations were conducted in the SPSS 23 program (SPSS INC., Chicago, IL, USA), adopting a significance level of 5%.

## Results

**Fig. 2:**
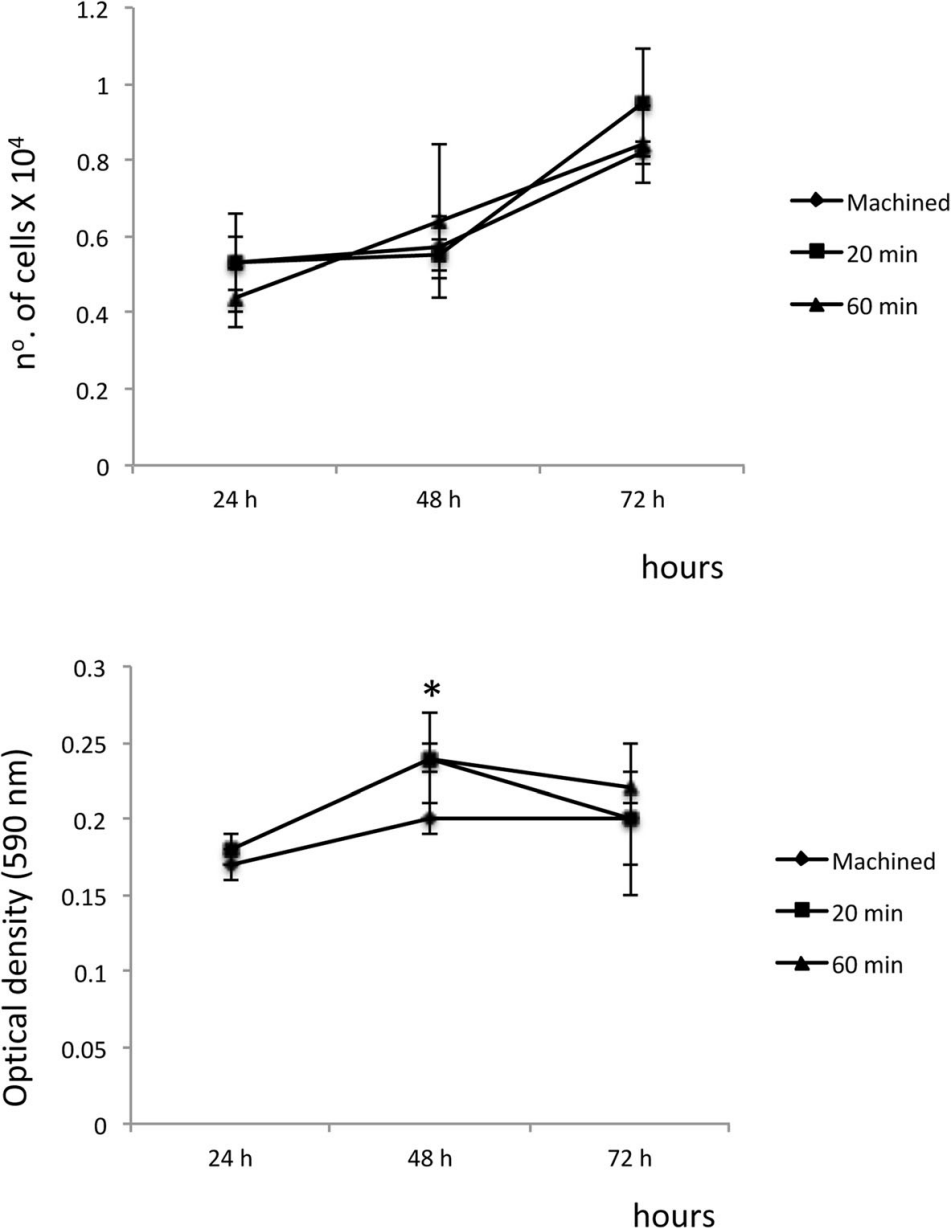
**a** Cell proliferation in gingival fibroblasts at 24 h, 48 h, and 72 h. The line chart represents the means and standard deviations from three separate experiments. **b** Cell viability assay in gingival fibroblast at 24 h, 48 h, and 72 h. The line chart represents the means and standard deviations from three separate experiments. The values are expressed in means (± SD). The asterisk symbol indicates a significant difference between groups (*p* < 0.05)

The results showed that acid treatment of the titanium surfaces for 20 or 60 min did not affect cell proliferation, either after 24 h (*p* = 0.484), 48 h (*p* = 0.698), or 72 h (*p* = 0.287) (Fig. 2a).

For the cell viability test, loss of viability was observed for the machined surface after 48 h of culture compared to the groups submitted to acid treatment for 20 or 60 min, which did not differ statistically between each other (Fig. 2b). No significant difference between groups regarding cell viability was verified after 24 h (*p* = 0.590) and 72 h (*p* = 0.799).

**Fig. 3:**
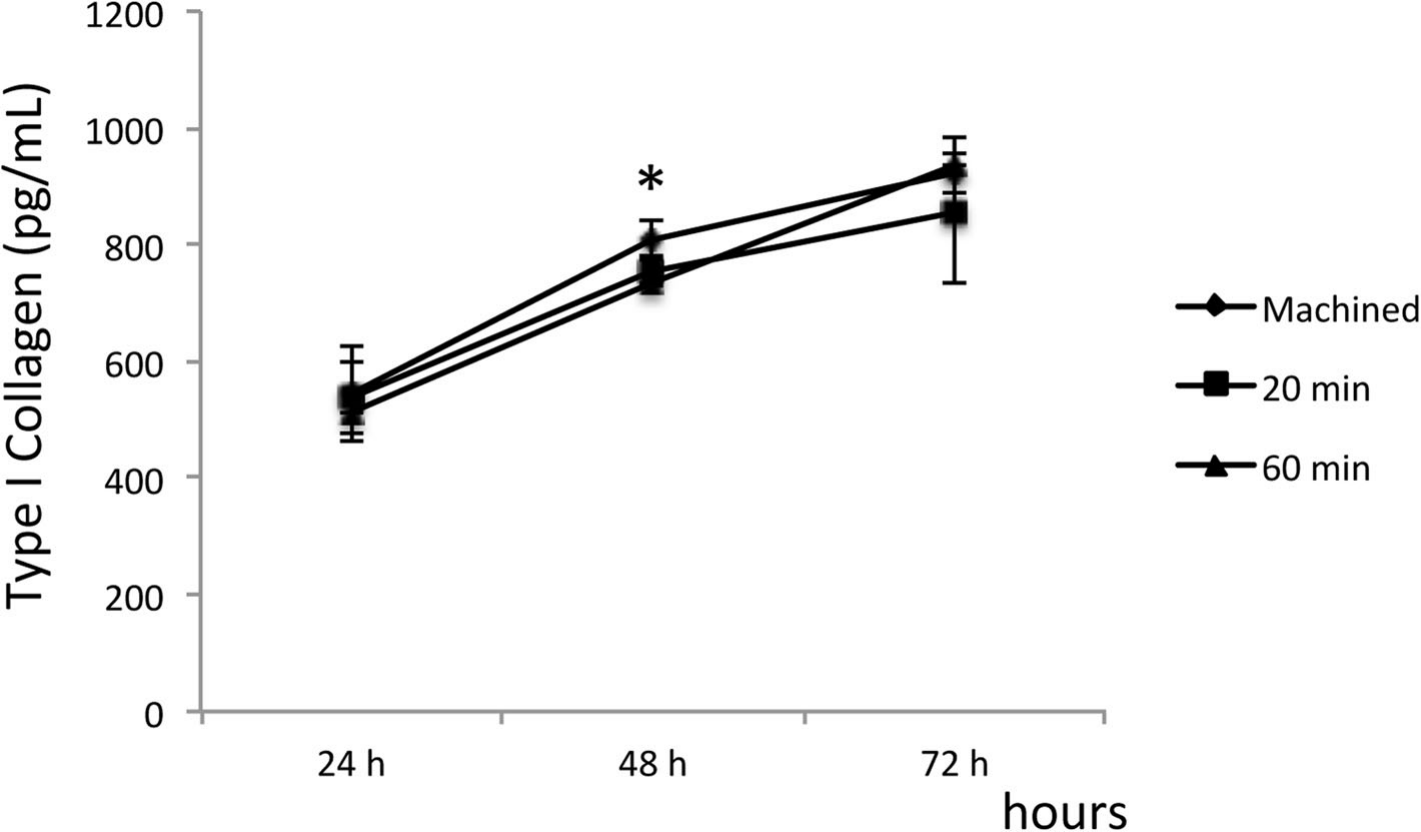
Quantification of type I collagen via ELISA. Data representative of the experiment run in triplicate. Values are expressed as mean (± SD). The asterisk symbol indicates a significant difference between groups (*p* < 0.05)

As for type I collagen synthesis, the lowest yields were observed on acid-treated surfaces, regardless of the application times (20 min or 60 min) after 48 h (Fig. 3). One-way ANOVA showed no significant difference between the groups regarding the expression of type I collagen at 24 h and 72 h.

## Discussion

Implant dentistry has for years focused on studying the interface between the bone and implant, searching for a suitable seal to prevent the advance of perimplant disease. Long-term prognosis of dental implants, however, depends not only on osseointegration, but also on the quality of the seal between the mucosa and the implant abutment [13].

The seal provided by the contact between mucosal tissue and the prosthetic abutment seems to be the safest route to implant longevity and long-term tissue stability. Currently, prosthetic abutments have their transmucosal collars manufactured with machined and polished surfaces. In the search for a titanium surface that favors cellular events, this study aimed to analyze if titanium surfaces treated with acid solution for different lengths of time could alter the behavior of fibroblasts, seeking a more stable union and promoting a better seal for perimplant health than otherwise polished surfaces.

The results of the present study showed that, in general, different acid treatment times did not increase the cellular events evaluated, namely cell proliferation, viability, and type I collagen secretion.

Some studies have reported that gingival fibroblasts seeded onto treated surfaces show greater proliferation and cellular viability when compared to machined surfaces [8, 9], indicating an advantage to treating the prosthetic abutment or the cervical portion of the implant to enhance a biological seal. It is important to highlight from the literature that cell type and cell cycle phase as well as surface type and chemical composition of the material may differ, which may in turn generate different results.

In spite of the numerous current options for prosthetic abutment materials given the high esthetic demand, titanium was used in the present study, which is a widely cited material in the literature as the gold standard when used as a prosthetic abutment, due to its high biocompatibility [14, 15]. Thus, the effect of applying an acid treatment protocol, which is widely used for dental implant surface treatment, on the proliferation, synthesis, and secretion of collagen by gingival fibroblasts was studied. The results obtained from the acid treatments allowed for smooth and minimally rough surfaces, according to the criteria adopted by Albrektsson and Wennergerg [16], respectively, for the acid treatments after 20 min and 60 min. This indicates that, despite acid treatment, 20 min was not sufficient to create surface roughness on the titanium surface with the acid solutions used.

The results showed that, regardless of the treatment used, there was no difference in the values of cell proliferation in the different times of analysis. Similarly, cell viability values did not differ, especially after 24 h and 72 h. Corroborating the results of this study, Baltriukiene et al. [17] used grade 2 titanium disks with different surface treatments and demonstrated that the modified titanium surface did not affect human gingival fibroblast viability. Interestingly, some studies show that smooth surfaces increase the proliferation and adhesion of fibroblasts [18, 19], which raises the question of whether the recommended acid treatment of titanium surfaces indeed has an advantage in terms of promoting greater biological sealing. In addition, increased surface roughness promotes bacterial colonization and increased retention [20, 21], contributing to increased biofilm formation.

In the present study, human gingival fibroblasts were used in a similar strategy as per other reports in the literature [10, 15, 22–25]. In spite of *in vivo* studies representing the ideal environment to investigate tissue responses, *in vitro* studies facilitate the investigation of a specific and isolated factor of the tissue response [26]. In addition to being widely used in cell biocompatibility studies, considering the various topographic surface features, fibroblasts are responsible for the biosynthesis of collagen and other extracellular matrix molecules.

To establish the amount of protein secreted by fibroblasts, we chose type I collagen, as it is the most abundant protein in the extracellular matrix of gingival connective tissue, representing almost 90% of the total organic matrix in mature bone. The results revealed differences between groups only at 48 h of culture, where the lowest titers were observed when surfaces were treated with acid solutions. At 24 h and 72 h, no significant difference was observed regarding collagen secretion, though the highest quantification occurred at 72 h for the surfaces treated for 60 min. These results are in agreement with the findings by Ramaglia et al. [9] and Velasco-Ortega et al. [24], in which they suggested that roughened surfaces may improve the biological behavior of fibroblasts as well as the process of perimplant soft tissue healing and osseointegration compared to machined surfaces.

The connective tissue seal around the abutment is crucial for perimplant health. Several efforts have previously been made to optimize abutment surfaces, though without consensus on the ideal surface features. Blasquez et al. [11] have shown in their systematic review that different types of surface modifications for implant abutments may provide benefit to the surrounding connective tissue, which generally corroborate the results reported herein. It is noteworthy that different methodologies and cell types, together with different types of acid solutions, might lead to different outcomes compared to the present study.

Cao et al. [25] with the aim of investigating the effects of different decontamination treatments on the microstructure of titanium surfaces as well as the proliferation and adhesion of human gingival fibroblasts showed that proliferation and adhesive strength were higher in the machined surfaces than on treated surfaces. These results led the group to conclude that proliferation and adhesion increases as surface roughness decreases.

## Conclusions

Therefore, the results of the present study indicate that the acid treatments used did not compromise cell growth nor collagen synthesis by gingival fibroblasts compared to machined titanium. The cost-benefit of manufacturing prosthetic abutments with treated surfaces should however be considered. In addition, *in vitro* studies using human keratinocytes and histological studies *in vivo* should be performed to identify the profile and direction of the fibers associated to these surfaces, which may shed a light on whether the appropriate perimplant biological seal actually occurs. The literature shows a lack of conclusive studies on this subject, so further work is needed to reach an ideal surface for prosthetic components over implants, promoting perimplant connective tissue health based on tissue adhesion and, consequently, a better seal between soft tissue and prosthetic component.

## Data Availability

All data generated or analyzed during this study are included in this published article.
